# Cohort Profile: The 2015 Pelotas (Brazil) Birth Cohort Study

**DOI:** 10.1093/ije/dyx219

**Published:** 2017-11-03

**Authors:** Pedro C Hallal, Andréa D Bertoldi, Marlos R Domingues, Mariângela Freitas da Silveira, Flávio F Demarco, Inácio Crochemore M da Silva, Fernando C Barros, Cesar G Victora, Diego G Bassani

**Affiliations:** 1 Post-Graduate Program in Epidemiology, Federal University of Pelotas, Pelotas, Brazil; 2 Post-Graduate Program in Physical Education, Federal University of Pelotas, Pelotas, Brazil; 3 Post-Graduate Program in Dentistry, Federal University of Pelotas, Pelotas, Brazil; 4 Centre for Global Child Health, Hospital for Sick Children & Department of Paediatrics, University of Toronto, Toronto, ON, Canada

## Why was the cohort set up?

Pelotas is a Southern Brazilian city with a current population of 344 000 inhabitants. Children delivered in the city’s hospitals in 1982, 1993 and 2004 and their mothers, were the target population for three birth cohort studies.^1–3^ Subjects from the cohorts are still being followed up every few years,^1–3^ constituting what is probably the largest set of birth cohorts in the same geographical location in low- or middle-income countries.

Continuing with the regular 11-year intervals, the 2015 cohort was launched to provide detailed information on time trends in maternal and child health, behaviours, nutrition, development and related factors. It also allows the study of time trends in socioeconomic and other types of health inequalities, and of how exposure-outcome associations are changing over time during a period of rapid epidemiological and nutritional transitions.

## Who is in the cohort?

**Figure 1:**
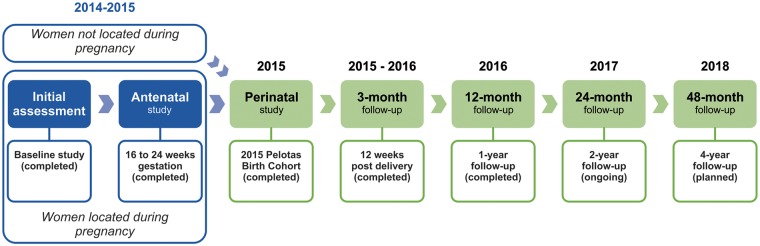
2015 Birth Cohort recruitment and follow-up schedule.

The 2015 cohort differed from the previous studies by attempting to recruit pregnant women during antenatal care, rather than soon after delivery, thus allowing for the prospective collection of pregnancy-related variables. This parallel antenatal clinic study enrolled 73.8% of the mothers who subsequently delivered children included in the cohort. To maintain consistency, from the perinatal study phase onwards (Figure 1) we adopted the same recruitment strategies used in the three previous cohorts. Over 99.0% of all births in the city have taken place in health facilities since the mid 1900s. All hospital-delivered children born between 1 January and 31 December 2015 were eligible for inclusion as long as their mothers lived in the urban area of Pelotas as defined in the first birth cohort (1982).^1^ The catchment area for the earlier cohorts included a small fishing village that is no longer classified as urban, and an isolated urban area that was later annexed to the neighbouring city of Capão do Leão. Births from these two areas were included in the 2015 cohort to maintain comparability with the earlier studies. No new urban areas have sprung up since 1982 outside the city limits, as population growth was modest, at 1.2% a year.

Fixed research teams were stationed daily at the four hospitals where 99.9% of all deliveries took place. A fifth hospital where the remaining births occurred was visited daily by a mobile team. Most mothers of eligible children were interviewed up to 1 day (96.7% of interviews) or up to 2 days after delivery (99.2%). A standardized computer-assisted questionnaire was used for the interviews. The study protocol was reviewed and approved by the School of Physical Education Ethics Committee at the Federal University of Pelotas (CAAE registration number: 26746414.5.0000.5313). The bio-repository was approved by School of Medicine Ethics Committee at the Federal University of Pelotas (CAAE registration number: 38976214.0.0000.5317). This cohort is registered at [clinicaltrials.gov] under the number NCT03271723. The sub-studies within this cohort are registered under the numbers NCT02148965 (PAMELA Trial) and NCT02788630 (Sleep Trial). Written informed consent was obtained from mothers identified during pregnancy, and from parents or guardians at every visit after delivery. In spite of population growth, fertility decline was intense; there were 6011 total births in 1982 compared with only 4389 in 2015.

## How often have they been followed up?

As of October 2017, the cohort has been followed up four times; the ongoing 24 months’ visit started in January 2017. Figure 1 describes the follow-up visits planned from pregnancy to age 4 years. Our experience with the previous cohorts advised against visiting sub-samples for measuring the main outcomes under study, as this may lead to a small subset of subjects with complete information. Follow-up visits thus, covered the full cohort, even if this led to an increase in the time intervals between visits due to funding and logistical limitations.

### Antenatal component

The 123 health facilities and private clinics providing antenatal care in the city were visited or contacted weekly, between May 2014 and December 2015, to identify pregnant women expected to give birth during 2015. These women were visited at home or invited to the research clinic between 16 and 24 weeks of gestation to answer a health questionnaire and receive an accelerometer to be worn for 7 days. Three types of questionnaires were developed and applied according to the gestational age of the woman at enrolment. Those before 16 weeks of pregnancy answered the initial assessment questionnaire, and were contacted again at 20 weeks (range 16–24 weeks) to answer the main assessment questionnaire. Women who were enrolled after 16 weeks of gestation responded to a combined assessment questionnaire, integrating the two initial assessment and main assessment questionnaires. The content of each of the three questionnaires is described in Table 1.

**Table 1.** Contents of the questionnaires and data collection tools, Pelotas 2015

Modules	Pre-natal assessments*			Post-natal assessments			
	Initial	Main	Combined	Peri-natal	3-months	12-months	24-months
**Eligibility Criteria**	X	X	X	X			
**Identification/Contact information**	X	X	X	X	X	X	X
**Socio-demographic characteristics**	X	X	X	X	X	X	X
**Maternal characteristics**	X		X	X			
**Pre-natal care**	X	X	X	X			
**Pregnancy card (scanned image)**^#^	X	X	X	X			
**Reproductive health history/contraception**				X	X	X	X
**Pre-pregnancy health**	X		X	X			
**Medicine use**	X	X	X	X^m^	X	X	X
**Paternal characteristics**	X		X	X			
**Physical activity questionnaire**	X	X	X	X	X	X	X
**Wellbeing**		X	X				
**Alcohol use**		X	X	X			
**Tobacco use**	X	X	X	X	X	X	X
**Illicit drug use**		X	X	X			
**Employment assessment**				X^m^		X	X
**Oral health**		X	X			X	X
**Pregnancy health**	X	X	X	X			
**Post-partum care**					X^m^^,^^c^		
**Edinburgh Postnatal Depression Scale**		X	X	X	X	X	X
**Pregnancy card image obtained**^#^	X	X	X	X			
**Obstetric abuse**					X		
**Anthropometry**				X^c^	X^m^^,^^c^	X^m^^,^^c^	X^m^^,^^c^
**Breastfeeding/Complementary feeding**				X	X	X	X
**Physical Activity (Accelerometry)**						X^p^^,^^c^	X^m^^,^^c^
**Sleep Actigraphy**						X	X
**Sleep Characteristics**					X	X	X
**Child Development (Oxford-NDA)**						X	X
**Child Health/immunization**				X	X	X	X
**Child morbidity – health-care use**					X	X	X
**Child-care arrangements**						X	X
**Child screen-time**						X	X
**Health expenditures**					X	X	X
**Saliva sample (DNA)**							X

* Pre-natal assessment instruments varied depending on the GA at enrollment. Women identified and enrolled before 16 weeks pregnancy answered the ‘initial assessment’ questionnaire and between weeks 17 and 24 a ‘main assessment’ questionnaire was applied (ideally at week 20 of pregnancy). Women enrolled after 16 weeks responded to the ‘combined assessment’ that consisted of a combination of the information collected in the ‘initial assessment’ and ‘main assessment’. All pre-natal assessment instruments collect information pertaining to the women and their partners.

m Maternal; ^p^Paternal; ^c^Child.

# Pregnancy card contains information on laboratory results, clinical assessments, vaccines, medicines taken during pregnancy, family health history, last menstrual period, estimated delivery date based on LMP and based on US, estimated gestational age based on LMP and ultrasounds (at three time points during pregnancy).

**Table 2.** Characteristics of mothers and children enrolled in the different stages of the 2015 Pelotas Birth Cohort

Variable	Cohort members included in the antenatal study		Cohort members		Cohort members included in the 3-month follow-up		Cohort members included in the 12-month follow-up	
	N	% (95%CI)	N	% (95%CI)	N	% (95%CI)	N	% (95%CI)
Maternal age (years)								
< 19	283	8.9 (7.9 – 9.9)	431	10.1 (9.2 – 11.0)	416	10.1 (9.2 – 11.1)	409	10.2 (9.3 – 11.2)
19 – 34	2432	76.0 (74.5 – 77.5)	3210	75.1 (73.8 – 76.4)	3091	75.2 (73.9 – 76.5)	3017	75.1 (73.7 – 76.4)
≥ 35	484	15.1 (13.9 – 16.4)	633	14.8 (13.8 – 15.9)	602	14.7 (13.6 – 15.8)	592	14.7 (13.7 – 15.7)
Maternal education (years complete of schooling)								
Zero	5	0.2 (0.1 – 0.4)	17	0.4 (0.2 – 0.6)	17	0.4 (0.2 – 0.7)	16	0.3 (0.2 – 0.6)
1 – 4	224	7.0 (6.2 – 7.9)	374	8.8 (7.9 – 9.4)	351	8.5 (7.7 – 9.4)	341	8.5 (7.7 – 9.4)
5 – 8	740	23.2 (21.7 – 24.6)	1095	25.6 (24.3 – 27.0)	1058	25.8 (24.4 – 27.1)	1023	25.5 (24.1 – 26.8)
≥ 9	2228	69.7 (68.1 – 71.2)	2787	65.2 (63.8 – 66.6)	2682	65.3 (63.8 – 66.7)	2636	65.6 (64.2 – 67.1)
Wealth index (quintiles)								
Poorest	515	16.6 (15.4 – 18.0)	824	20.0 (18.8 – 21.2)	797	20.1 (18.8 – 21.3)	759	19.5 (18.3 – 20.8)
Second	623	20.1 (18.7 – 21.5)	829	20.1 (18.9 – 21.3)	801	20.2 (18.9 – 21.4)	787	20.3 (19.0 – 21.6)
Third	652	21.0 (19.6 – 22.5)	820	19.9 (18.7 – 21.1)	789	19.9 (18.6 – 21.1)	781	20.1 (18.9 – 21.4)
Fourth	651	21.0 (19.6 – 22.5)	823	19.9 (18.8 – 21.2)	789	19.9 (18.6 – 21.1)	780	20.1 (18.9 – 21.4)
Wealthiest	660	21.3 (19.9 – 22.8)	831	20.1 (18.9 – 21.4)	797	20.1 (18.8 – 21.3)	776	20.0 (18.8 – 21.3)
Family income (minimum wages)								
≤ 1	297	9.8 (8.8 – 10.9)	498	12.4 (11.4 – 13.4)	477	12.3 (11.3 – 13.4)	465	12.3 (11.3 – 13.4)
1 – 3	1446	47.8 (46.0 – 50.0)	1891	47.1 (45.5 – 48.6)	1824	47.2 (45.6 – 48.7)	1783	47.1 (45.5 – 48.7)
4 – 6	841	27.8 (26.2 – 29.4)	1064	26.5 (25.2 – 27.9)	1031	26.7 (25.3 – 28.1)	1013	26.7 (25.4 – 28.2)
7 – 9	251	8.3 (7.4 – 9.3)	307	7.6 (6.9 – 8.5)	290	7.5 (6.7 – 8.4)	285	7.5 (6.7 – 8.4)
≥ 10	192	6.3 (5.5 – 7.3)	256	6.4 (5.7 – 7.2)	247	6.4 (5.7 – 7.2)	241	6.4 (5.6 – 7.2)
Parity								
1	1656	51.8 (50.1 – 53.5)	2136	50.0 (48.5 – 51.5)	2056	50.0 (48.5 – 51.7)	2010	50.1 (48.5 – 51.6)
2	1001	31.3 (29.7 – 32.9)	1320	30.9 (29.5 – 32.3)	1273	31.0 (29.6 – 32.4)	1248	31.1 (29.7 – 32.5)
≥ 3	540	16.9 (15.6 – 18.2)	817	19.1 (18.0 – 20.3)	780	19.0 (17.8 – 20.2)	758	18.9 (17.7 – 20.1)
Physical activity practice at 1st trimester of pregnancy								
Yes	410	12.8 (11.7 – 14.0)	507	11.9 (10.9 – 12.8)	491	12.0 (87.0 – 89.0)	480	12.0 (11.0 – 13.0)
No	2787	87.2 (86.0 – 88.3)	3766	88.1 (87.1 – 89.1)	3618	88.0 (11.0 – 13.0)	3536	88.0 (87.0 – 89.0)
Type of delivery								
Normal	1042	32.6 (31.0 – 34.2)	1489	34.8 (33.4 – 36.3)	1443	35.1 (33.7 – 36.6)	1408	35.0 (33.6 – 36.5)
C-section	2157	67.4 (65.8 – 69.0)	2786	65.2 (63.7 – 66.6)	2667	64.9 (63.4 – 66.3)	2610	65.0 (63.5 – 66.4)
Sex								
Male	1622	50.7 (49.0 – 52.4)	2164	50.6 (49.1 – 52.1)	2077	50.5 (49.0 – 52.1)	2044	50.9 (49.3 – 52.4)
Female	1577	49.3 (47.6 – 51.0)	2111	49.4 (47.8 – 50.9)	2033	49.5 (47.9 – 51.0)	1974	49.1 (47.6 – 50.7)
Birth weight (g)								
< 2500	307	9.6 (8.6 – 10.7)	428	10.1 (9.2 – 11.0)	391	9.5 (8.7 – 10.5)	378	9.4 (8.5 – 10.4)
2500 – 3499	2040	63.9 (62.2 – 65.6)	2697	63.3 (61.9 – 64.8)	2615	63.7 (62.2 – 65.1)	2558	63.7 (62.2 – 65.2)
≥ 3500	845	26.5 (25.0 – 28.0)	1133	26.6 (25.3 – 28.0)	1101	26.8 (25.5 – 28.2)	1079	26.9 (25.5 – 28.3)
**Total**	**3199**		**4275**		**4110**		**4018**	

### Perinatal component

During 2015, all live and stillbirths occurring in the five hospitals were identified. The mothers of live-born infants were invited to join the cohort. After signing the informed consent form, mothers were interviewed about their health and behaviours during pregnancy and delivery (Table 1). Newborn anthropometry was carried out by our research team following the protocol used in the previous cohorts. Length was measured using the Harpenden Infantometer (Chasmors, UK) with 1 mm precision, weight was measured using the SECA portable paediatric scales model 376 (SECA, Germany) and head circumference was measured using CARDIOMED WCS measuring tape (CARDIOMED, China) with 1 mm precision.

A system put in place to revert refusals was able to revert 37 of the original 88 refusals (42.0%) by having a senior study team member meet with the families to explain the importance and purposes of the study in detail.

### Three and 12 months’ follow-up

Home visits were carried out at the ages of 3 and 12 months. In special situations, interviews were conducted at our research centre (e.g. when the family had moved out of Pelotas but agreed to come to the city for assessment). During these visits, parents/caregivers answered a questionnaire (Table 1) and both mothers and children had anthropometric measures taken. At the 3-month visit, mothers were weighed using TANITA UM80 scales (TANITA, Japan) with 100 g precision, and were measured using a portable aluminum stadiometer with 1-mm precision manufactured for this study. Infants had their length measured using a SANNY ES2000 portable anthropometer (SANNY, Brazil) with 5 mm precision and were weighed using a portable electronic scale with 10 g precision. At the 12-month follow-up, children were weighed on their mothers’ laps using SECA 803 scales (SECA, Germany) with 100 g precision; the mother then handed the child to someone else and her weight was obtained. The child’s weight was provided automatically by the scale as the difference between the two weights. Children were weighed naked and mothers were using light clothes that were recorded in the questionnaire; clothes’ weights were discounted from the woman’s total weight. Maternal height and child length were assessed using the same instruments used in the 3-month visit. The child’s father entered a 7-day accelerometry study at the 12-month follow-up.

### 24-months’ follow-up

The 24-months’ follow-up study started in January 2017 and will last until December 2017. This follow-up study updates information collected on the previous visit, collects information on physical activity from the mothers using accelerometry and also collects four nights of accelerometry data to assess the children’s sleep patterns. In addition, saliva samples are being obtained from all cohort members, to extract genetic material.

### Future follow-up visits

Between January and December 2019, the children are going to be visited as part of the 4-year follow-up study. If funding is obtained, further visits are planned to take place at ages of 7, 11, 15 and 18 years.

## What has been measured?

The 2015 cohort was designed to collect data using protocols and procedures that are aligned with the previous cohorts, but to also expand the breadth of data collected on selected areas. In common with the earlier studies, the following information was collected:

- pre-pregnancy health status, antenatal care characteristics (number and timing of visits), illnesses during pregnancy, gestational age (last menstrual period and ultrasound), health behaviours and socio-demographic characteristics during pregnancy;
- maternal health characteristics during pregnancy and during the first year after delivery, including anthropometry, morbidity, mortality, mental health, contraception and health care use;
- information on previous births (number, survival, birthweight, type of delivery);
- fetal (including stillbirths), neonatal and post-neonatal deaths, with cause of death ascertainment and mortality audits;
- morbidity and nutritional outcomes during infancy, including growth, breastfeeding, neurocognitive development, infections and injuries;
- health services use during infancy, including hospitalizations, immunizations, uses of medicines by the mothers (during and after pregnancy) and children (at 3 and 12 months), and routine and curative outpatient contacts;
- risk factors associated with the outcomes listed above, including health care, socioeconomic, demographic, behavioural, paternal and environmental characteristics;
- socioeconomic, ethnic and gender inequalities in outcomes and in access to and use of health care, to identify high-risk groups;
- genetic material from children (saliva samples being collected during the 24-month follow-up).

In addition to the above variables, the 2015 cohort has expanded data collection in three aspects. First, recruitment during pregnancy enabled the prospective collection of detailed information on the characteristics of pregnant women and on antenatal care and conditions arising during gestation. Second, we carried out extensive investigations on physical activity by including parental and child accelerometry, measured with ActiGraph accelerometers*,* model *wGT3X-BT* (ActiGraph, USA). Third, children were subjected to neurodevelopmental assessment at age 12 months using the Oxford Neurodevelopmental Assessment tool (OX-NDA) that is being validated within this cohort against the Bayley Scales of Infant and Toddler Development.^4^ At age 24 months, the children are being assessed using the INTERGROWTH-21st Project Neurodevelopment Package (INTER-NDA).^5^

### Sub-studies within the 2015 Pelotas Birth Cohort

In addition to the main visits to all children and women, three sub-studies and two small randomized controlled trials were nested in the cohort.

#### Perinatal and infant morbidity and mortality sub-study

All stillbirths (birthweight above 500 g or gestational age above 20 weeks) were identified, as well as all deaths of live-born infants during the first year of life. All death certificates registered in the city for children born in the cohort catchment area in 2015 were reviewed by the study team on a bi-weekly basis during 2015 and 2016. In addition, our research team conducted daily visits to all hospitals that admit children in Pelotas between 1 January and 31 December 2016 to collect information on admissions and deaths among cohort members. Admissions for phototherapy or newborn admissions due to maternal admissions were not included in the morbidity database.

#### Sub-study on use of medicines during the admission for delivery

A sub-study to evaluate the use of medicines during the admission for delivery included the review of hospital records of all 1409 deliveries between 5 June and 5 October 2015.

#### Oral health sub-study

The oral health sub-study identified 3125 eligible women and 15 trained dentists examined 3100 of these women (99.2%) during pregnancy. The women excluded from the oral health sub-study (*n* = 25) refused to participate. Of the women examined, 2496 delivered babies that are included in the perinatal study of the 2015 Pelotas Birth Cohort (80.1%). Clinical evaluations included the decayed, missing and filled teeth (DMFT) index, assessment of periodontal health and examination of oral soft tissues. Questionnaires collected information on oral hygiene and use of dental services during pregnancy. At 12 months, mothers provided information on the number of erupted teeth and on the use of oral health services by the infant. At 24 months, information was also collected on the child’s oral hygiene practices. The 48-month follow-up will include a clinical examination to assess cavities, occlusion issues, trauma and enamel development defects in the children.

### Randomized clinical trials nested in the 2015 Pelotas Birth Cohort

The cohort includes two nested randomized clinical trials.^6^ PAMELA (Physical Activity for Mothers Enrolled in Longitudinal Analysis) enrolled women between the 16th and 20th weeks of gestation. These were randomly allocated to either a control group (*n* = 426), who were advised to keep their usual daily activities, or an intervention group (*n* = 213) who were engaged in an exercise programme (aerobics, strength and flexibility training) consisting of three 60-min sessions weekly throughout pregnancy. Baseline data including blood and urine samples, anthropometry and pulmonary function were collected at enrolment, and assessments were repeated 8 and 16 weeks post-baseline. Maternal and child outcomes were measured at the 36th week of gestation, at birth and at 3 and 12 months postpartum. The trial participants will also be assessed at 24 and 48 months postpartum. The trial protocol is described in detail elsewhere.^7^

The Sleep Trial is a 1:1 parallel group single-blinded randomized controlled trial that enrolled a total of 552 infants at 3 months of age. The study protocol is described in detail elsewhere.^8^ Children were considered eligible if mothers reported that their infants slept, on average, for less than 15 h a day. After block randomization, the intervention group received home visits by trained fieldworkers who provided standardized advice on general practices to promote infant’s self-regulated sleep, as well as a booklet including content to aid the mother in implementing the intervention. Mothers in the intervention arm received reinforcement calls 2 days after the initial visit, and on day 3 the fieldworkers conducted a reinforcement visit. The main outcome being assessed is the change in the mean nighttime self-regulated sleep duration (the maximum amount of time the child stays asleep or awake without awakening the parents) at ages 6, 12 and 24 months, using actigraphy, activity diary records and questionnaires. Secondary outcomes include conditional linear growth between ages 3–12 and 12–24 months, as well as neurocognitive development at ages 12 and 24 months.

### Additional sub-studies

In addition to the studies described above, the 2015 Pelotas birth cohort is also being used to validate a series of new instruments/tools to collect information on diverse areas, including child development, parenting and use of accelerometry to measure sleep patterns in infants. Details of these studies will be available in the near future.

## What has it found?

As the study is still ongoing, limited early findings are described below.

The antenatal component of the cohort (pregnancy cohort) identified and enrolled 4426 pregnant women with an expected delivery date (EDD) between 15 December 2014 and 19 May 2016 (eligibility window). The EDD was estimated using self-reported date of the first day of last menstrual period (LMP), as well as the EDD recorded by the health care team in the National Pregnancy Card. If either EDD fell within the eligibility window, the woman was included in the antenatal study. A total of 2414 (54.5%) women were enrolled before 16 weeks of gestation and 2012 after 16 weeks of gestation (58.2% between 16 and 24 weeks, and 41.8% after 24 weeks), according to LMP at the time of interview. In total, 91.1% of the women enrolled during pregnancy reported being sedentary (not engaged in regular physical activity). Only 46.3% of pregnancies were planned and 43.4% of all women were primiparae. A total of 1277 women identified during the antenatal component were not enrolled in the cohort due to ineligibility at time of delivery (i.e. residing and/or delivering outside the cohort catchment area, not completing the pregnancy, delivering before 1 January 2015 or after 31 December 2015).

We were thus able to identify during pregnancy and interview 3155 (73.8%) of the mothers of liveborn children who were later enrolled in the cohort. Of these, 198 women were identified before 16 weeks of gestation, but were either not located for an interview during 16–24 weeks gestation, or refused to participate. A total of 99 who had not attended antenatal care, and therefore could not be captured by our antenatal study, were identified at delivery.

During pregnancy, 65.8% of the women indicated preference for vaginal delivery, but the proportion of women who delivered vaginally was 34.8% overall, and only 42.9% among those who indicated preference for vaginal delivery. Caesarean sections reached the alarming prevalence of 65.2% in 2015.

**Figure 2:**
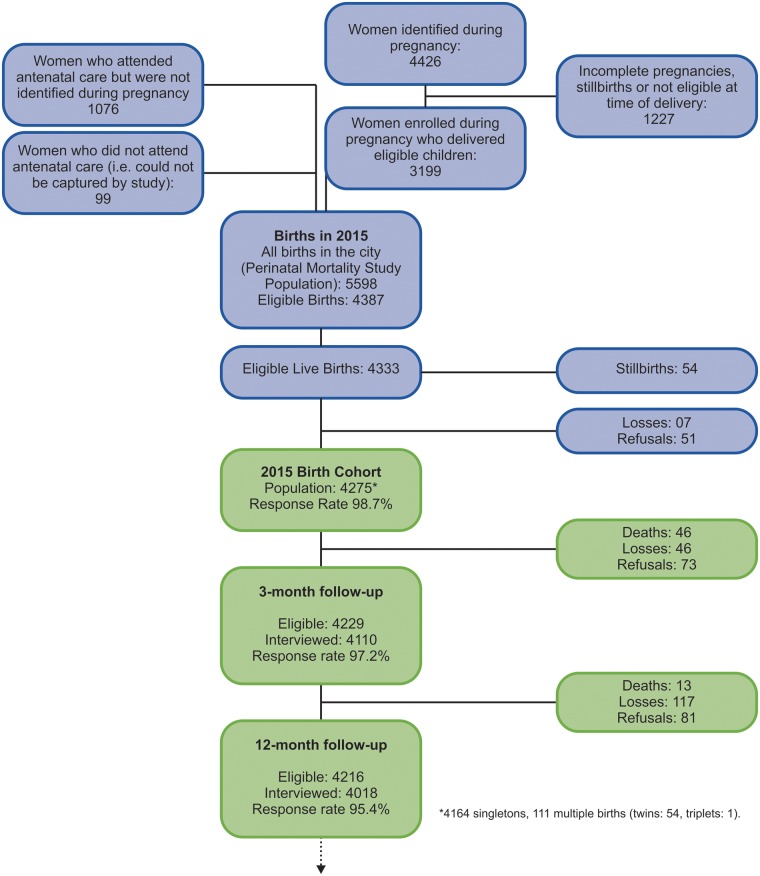
Study participant flowchart.

Figure 2 shows the flowchart describing enrolment and participation. Out of the 5598 children born in Pelotas during 2015, 4387 were born to mothers living in the urban area. Of these, 54 were stillborn and the remaining 4333 were the target population for our study. Of these, 4275 (98.7%) were effectively enrolled in the study and thus became the study population. There were 51 refusals and seven births that were not captured by the study teams in spite of daily visits to all hospitals.

The 3-month follow-up included 97.2% of the original birth cohort: a slight improvement in comparison with our 2004 cohort follow-up rate of 95.7%.^3^ The 12-month follow-up rate of 95.4% is also improved in comparison with 2004 (92.3%).^3^^,^^9^ Known deaths between birth and each follow-up are included in the numerator when calculating follow-up rates, as children who died are treated as having been located.

Comparisons with earlier cohorts show interesting trends. The proportions of teenage (< 20 years of age) pregnancies were 15.4% in 1982,^10^ 17.4% in 1993,^11^ 14.0% in 2004^3^^,^^9^ and 10.1% in 2015. Low birthweight (< 2500 g) prevalence has been relatively stable: 9.0% in 1982,^10^ 9.7% in 1993^11^ amd 10.0% in both 2004^3^^,^^9^ and in 2015. Maternal education continues to improve, with the mean number of years of schooling increasing from 6.5 in 1982^10^ to 6.7 in 1993,^11^ 8.1 in 2004^3^^,^^9^ and 10.1 in 2015. The proportion of births among women from families earning less than the minimum wage has decreased from 21.0% in 2004^9^ to 12.4% in 2015. The 12-month follow-up was able to interview 4018 children, which corresponds to a follow-up rate of 95.4% in comparison with 94.2% in 2004.^3^^,^^9^

## What are the main strengths and weaknesses?

The main strengths of the study include the fact that, for a large proportion of mothers, data on pregnancy exposures were collected during antenatal care, instead of retrospectively as in most birth cohorts. The instruments and methods used were consistent with the previous cohorts allowing comparability across cohorts, and the same core research team was in charge of the four studies over the 33-year period. Data collection methods improved over these three decades and new study areas were added, such as the use of accelerometry to measure sleep and physical activity objectively. Our experience with the previous cohorts also enabled us to reduce losses and refusals in the follow up visits.

## Can I get hold of the data? Where can I find out more?

Collaborations for the analysis of the data are welcome and we have ongoing collaborations with the Centre for Global Child Health at the Hospital for Sick Children in Toronto, Canada, the MRC Epidemiology Unit in Cambridge, UK, the Institute of Child Health in London, UK, the University of Oxford in Oxford, UK and the Norwegian School of Sport Sciences in Oslo, Norway. We encourage doctoral or post-doctoral fellows from other institutions to spend a period of time in Pelotas for such collaborations, as well as encourage our students or fellows to spend time in other institutions, thus helping build networks and strengthen skills and capacity. The 2015 Pelotas Birth Cohort is a supported access resource and to request access to the data, please follow the instructions at [http://www.epidemio-ufpel.org.br/data_access].

**Profile in a nutshell**

- The 2015 Pelotas Birth Cohort is the most recent of the four population-based prospective studies of the health and development of children in this Southern Brazilian city (population 344 000), now spanning over four decades.
- The 4275 children included in the cohort were born between 1 January and 31 December 2015, from mothers who lived in the urban areas of the city.
- The mothers of 73.8% of all cohort children were identified and followed up during pregnancy.
- Follow-ups took place at ages 3 and 12 months, and 4216 children remain eligible for the currently ongoing follow-up (24 months), with all follow-up rates being above 95%.
- Data were collected on sociodemographic characteristics, health-related behaviours, antenatal health and health care, physical activity (self-reported and by actigraphy), delivery and perinatal conditions, child anthropometry, child sleep patterns, child mortality and morbidity, breastfeeding and dietary patterns, neurocognitive development, access to health services and use of medicines by the mother and child. Genetic material on all children is being collected during the 24-month follow-up.
- The 2015 Pelotas Birth Cohort is a supported access resource[http://www.epidemio-ufpel.org.br/data_access].

## Funding

This article is based on data from the 2015 Pelotas (Brazil) Birth Cohort Study. This project is funded through a New Investigator Award (grant number 095582/Z/11/Z) from the Wellcome Trust to P.C.H. The study is conducted by the Postgraduate Program in Epidemiology of the Federal University of Pelotas, Brazil with the collaboration of the Brazilian Public Health Association (ABRASCO), and is currently also supported by the Brazilian National Research Council (CNPq) and the Coordination for the Improvement of Higher Education Personnel (CAPES) (grant number 2207/2012). D.G.B. was supported by CAPES and CNPq as a Special Visiting Scholar (Bolsista CAPES/BRASIL - grant number 036/2012 PVE).
